# Risedronate and Methotrexate Are High-Affinity Inhibitors of New Delhi Metallo-β-Lactamase-1 (NDM-1): A Drug Repurposing Approach

**DOI:** 10.3390/molecules27041283

**Published:** 2022-02-14

**Authors:** Ghazala Muteeb, Abdulrahman Alsultan, Mohd Farhan, Mohammad Aatif

**Affiliations:** 1 Department of Nursing, College of Applied Medical Science, King Faisal University, Al-Ahsa 31982, Saudi Arabia; graza@kfu.edu.sa; 2 College of Applied Medical Science, King Faisal University, Al-Ahsa 31982, Saudi Arabia; aalsultan@kfu.edu.sa; 3 Department of Basic Sciences, King Faisal University, Al-Ahsa 31982, Saudi Arabia; mfarhan@kfu.edu.sa; 4 Department of Public Health, College of Applied Medical Science, King Faisal University, Al-Ahsa 31982, Saudi Arabia

**Keywords:** antibiotic resistance, FDA-approved drugs, metallo-β-lactamase, molecular docking and simulation, structure-based drug design

## Abstract

Bacteria expressing New Delhi metallo-β-lactamase-1 (NDM-1) can hydrolyze β-lactam antibiotics (penicillins, cephalosporins, and carbapenems) and, thus, mediate multidrug resistance. The worldwide dissemination of NDM-1 poses a serious threat to public health, imposing a huge economic burden in the development of new antibiotics. Thus, there is an urgent need for the identification of novel NDM-1 inhibitors from a pool of already-known drug molecules. Here, we screened a library of FDA-approved drugs to identify novel non-β-lactam ring-containing inhibitors of NDM-1 by applying computational as well as in vitro experimental approaches. Different steps of high-throughput virtual screening, molecular docking, molecular dynamics simulation, and enzyme kinetics were performed to identify risedronate and methotrexate as the inhibitors with the most potential. The molecular mechanics/generalized Born surface area (MM/GBSA) and molecular dynamics (MD) simulations showed that both of the compounds (risedronate and methotrexate) formed a stable complex with NDM-1. Furthermore, analyses of the binding pose revealed that risedronate formed two hydrogen bonds and three electrostatic interactions with the catalytic residues of NDM-1. Similarly, methotrexate formed four hydrogen bonds and one electrostatic interaction with NDM-1’s active site residues. The docking scores of risedronate and methotrexate for NDM-1 were –10.543 kcal mol^−1^ and −10.189 kcal mol^−1^, respectively. Steady-state enzyme kinetics in the presence of risedronate and methotrexate showed a decreased catalytic efficiency (i.e., kcat/Km) of NDM-1 on various antibiotics, owing to poor catalytic proficiency and affinity. The results were further validated by determining the MICs of imipenem and meropenem in the presence of risedronate and methotrexate. The IC_50_ values of the identified inhibitors were in the micromolar range. The findings of this study should be helpful in further characterizing the potential of risedronate and methotrexate to treat bacterial infections.

## 1. Introduction

The discovery of penicillin and the development of other β-lactam antibiotics have revolutionized the healthcare sector’s ability to treat bacterial infections. The β-lactam antibiotics—such as penicillins, cephalosporins, carbapenems, and monobactams—are broad-spectrum and highly efficient antibiotics with low toxicity [1]. These features make them the drugs of choice in clinics to treat different kinds of bacterial infections. However, the overuse, misuse, and abuse of β-lactam antibiotics have led to the emergence of antibiotic resistance in bacteria. It is estimated that at least 700,000 deaths reported worldwide per year are directly related to drug-resistant pathogens [2]. The primary mechanism by which bacteria develop resistance against β-lactam antibiotics is the production of β-lactamases, which hydrolyze the amide bond of the β-lactam ring. Several bacteria carry multiple resistance markers and behave like “superbugs”, such as vancomycin-resistant *Enterococcus* (VRE), methicillin-resistant *Staphylococcus aureus* (MRSA), and New Delhi metallo-β-lactamase-1 (NDM-1). According to Ambler, β-lactamases are classified into four broad classes (A to D) on the basis of their molecular mechanism of action [3]. The class A, C, and D β-lactamases are known as serine β-lactamases, as they contain a serine residue at the active site. The class B β-lactamases use metal ions (usually Zn^2+^ ions) to hydrolyze β-lactam antibiotics, and are therefore known as metallo-β-lactamases (MBLs). On the basis of sequence similarity, MBLs are further divided into B1, B2, and B3 subclasses, amongst which the B1 subclass is the most clinically relevant. Moreover, another classification of β-lactamases on the basis of their functionality has been proposed by Bush et al. According to Bush’s classification, β-lactamases are classified into Group 1, comprising cephalosporinases; Group 2, including oxacillinases, penicillinases, extended-spectrum β-lactamases (ESBLs), and serine-based carbapenemases; and Group 3, containing metal-based carbapenemases [4]. Thus, NDM-1 belongs to subclass B1 according to Ambler’s classification, and Group 3 according to Bush’s classification of β-lactamases.

In 2009, the first case of NDM-1 was reported in India from a Swedish patient who acquired a urinary tract infection by *Klebsiella pneumoniae* [5]. Since then, NDM-1 and its mutants have spread globally, and pose a severe threat to human health, along with an enormous economic burden in the development of new antibiotics. NDM-1 can hydrolyze all of the β-lactam antibiotics—such as penicillins, cephalosporins, and carbapenems, which are considered to be the last resort of antibiotics [6]. Furthermore, bacteria expressing NDM-1 often carry genes for other resistance markers, such as quinolones, sulfonamides, rifampin, macrolides, aminoglycosides, and other serine β-lactamases, which make them multidrug-resistant bacteria, or “superbugs” [7]. The dissemination of NDM-1 has been reported in Gram-negative bacteria such as *Escherichia coli*, *K. pneumoniae*, *Enterobacter cloacae*, *Acinetobacter baumannii*, etc., in different hospital as well as domestic settings [8]. Over the years, several effective inhibitors of NDM-1—such as captoprils, thiol compounds, sulfanilamides, boric acid derivatives, natural compounds, etc.—have been reported in the literature [9,10,11,12,13]. However, none of the NDM-1 inhibitors have been approved for clinical use, due to concerns about their specificity, safety, and physiological properties. Although FDA-approved inhibitors such as clavulanic acid, sulbactam, and tazobactam are available to inhibit serine β-lactamases, they are unable to inactivate MBLs, including NDM-1 [14]. It is feared that there will be no antibiotics available in the future to overcome bacterial infections caused by multidrug-resistant (MDR) and extensively drug-resistant (XDR) pathogens. Thus, there is an urgent need to develop new antibiotics or identify novel inhibitors of β-lactamases in order to protect the existing β-lactam antibiotics.

In this study, we screened a library of FDA-approved drugs as potential inhibitors of NDM-1. We employed a variety of computational approaches, such as high-throughput virtual screening, molecular docking, molecular dynamics simulation, and free energy calculations. Furthermore, the potential of the identified inhibitors was validated by in vitro enzyme kinetics.

## 2. Results

### 2.1. Validation of Molecular Docking

**Figure 1:**
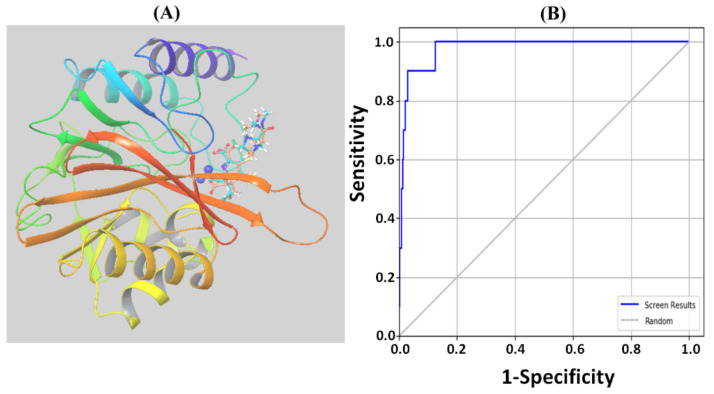
Validation of the molecular docking protocol: (**A**) Re-docking of the ligand present in the X-ray crystal structure to the active site of NDM-1, and calculation of the root-mean-square deviation (RMSD) between the docked pose and the crystal structure pose of the ligand. The RMSD between the two poses was 0.7634 Å. (**B**) Receiver operating characteristic (ROC) curve displaying the sensitivity and specificity of the docking mode.

The validity of the adopted molecular docking protocol was accessed by re-docking the ligand present in the X-ray crystal structure at the active site of NDM-1, and calculating the RMSD between the docked pose and the crystal structure pose (Figure 1A). The RMSD between the docked and crystal structure poses was estimated to be 0.7634 Å, suggesting a native-like binding pattern of the ligand. Furthermore, the enrichment calculations were performed on the highest scoring drug molecules with the highest negative docking scores. The receiver operating characteristic (ROC) curve was drawn between the sensitivity and selectivity to analyze the ability of the docking protocol to distinguish the active molecules from a set of inactive decoys (Figure 1B). The main advantages of using ROC curves over other conventional enrichment methods are that (1) they are not dependent on the number of actives in the test set, and (2) they consider sensitivity as well as specificity [15]. An ROC curve is a curve in which the number of actives found in the top ranked ligands is plotted as a function of the total number of ligands in the dataset. The fractional area under the ROC curve is known as the AUC (area under the curve). The ROC, BEDROC, and AUC values of the adopted molecular docking protocol were estimated to be 0.98, 0.758, and 0.97 respectively. Usually, the ROC values vary in the range of 0 to 1, with a value closer to 1 meaning a greater ability to differentiate the actives from the decoys. The AUC value of 0.97 indicated that there was a 97% better chance of randomly picking an active ligand than a decoy. The AUC was significantly higher than 0.5, indicating better suitability of the docking protocol than random discrimination in screening a large database to identify potential inhibitors of NDM-1 [16].

### 2.2. Analysis of Virtual Screening

In this study, an FDA-approved library of 2685 drugs was screened to identify novel inhibitors of NDM-1. The library was first screened by HTVS, followed by SP and then more stringent XP docking modes [17]. In HTVS, 825 drugs were found to have a binding affinity towards NDM-1 (Supplementary Table S1). These 825 drugs were further screening by SP docking, wherein the drug molecules were ranked after post-docking minimization of protein–ligand complexes. We identified 119 drug molecules with docking score energies in the range of −4.789 to −9.123 kcal mol^−1^ to form a docked complex with NDM-1. The Glide g-score of these drugs was in the range of −5.103 to −9.129 kcal mol^−1^, while the Glide e-model score varied in the range of −43.018 to −140.610 kcal mol^−1^ (Supplementary Table S2). The drug molecules with a docking score of ≤−8.0 kcal mol^−1^ in SP docking were further subjected to a more stringent XP docking (Table 1). The docking score, Glide g-score, and Glide e-model score of these selected drug molecules were in the range of −5.329 to −10.355 kcal mol^−1^, −5.329 to −10.543 kcal mol^−1^, and −26.326 to −80.917 kcal mol^−1^, respectively. The top scoring drugs with a docking score of ≤−9.0 kcal mol^−1^—namely, risedronate, methotrexate, pamidronate disodium, fludarabine, alendronate sodium, etidronate, and ibandronate sodium—were shortlisted for further analysis by MM/GBSA.

### 2.3. Free Energy Estimation by MM/GBSA

The free energy of interaction between NDM-1 and the shortlisted drug molecules was determined using the MM/GBSA approach, as reported previously [18]. The free energy of interaction (Δ*G_Bind_*) between NDM-1 and drugs was calculated by subtracting the individual free energies of the NDM-1 (*G_Protein_minimized_*) and drug molecules (*G_Ligand_minimized_*) from the free energy of the NDM-1-drug complex (*G_Complex_minimized_*). Overall, the following equation was used to compute different thermodynamic parameters related to binding free energy calculations:

$$\Delta G_{Bind}=\Delta G_{Coulomb}+\Delta G_{vdW}+\Delta G_{Covalent}+\Delta G_{H-bond}+\Delta G_{Lipo}+\Delta G_{Solv\_GB}+\Delta G_{Packing}$$

where Δ*G_Coulomb_*, Δ*G_vdW_*, Δ*G_Covalent_*, Δ*G_H-bond_*, Δ*G_Lipo_*, Δ*G_Sol_GB_*, and Δ*G_Bind_* represent the Coulomb energy, van der Waals’ energy, covalent binding energy, energy due to H-bonds, lipophilic energy, polar solvation energy, and Gibbs free energy respectively.

It is clear from Table 2 that binding free energies of different drug molecules were in the range of −19.25 to −44.16 kcal mol^−1^. The top two drug molecules—namely, methotrexate and risedronate—showed the lowest binding free energies, corresponding to −44.16 and −36.51 kcal mol^−1^, respectively. Further dissection of the binding free energy revealed that the NDM-1 and drug complexes were stabilized primarily by Coulombic (Δ*G_Coulomb_*) and van der Waals’ (Δ*G_vdW_*) energies, along with small contributions by hydrogen bond (Δ*G_H-bond_*), packing (Δ*G_Packing_*), and lipophilic (ΔG*_Lipo_*) energies. Conversely, polar solvation (Δ*G_Sol_GB_*) and covalent (Δ*G_Covalent_*) energies destabilized the NDM-1 and drug complexes.

### 2.4. Analysis of Molecular Docking

#### 2.4.1. Risedronate–NDM-1 Interaction

**Figure 2:**
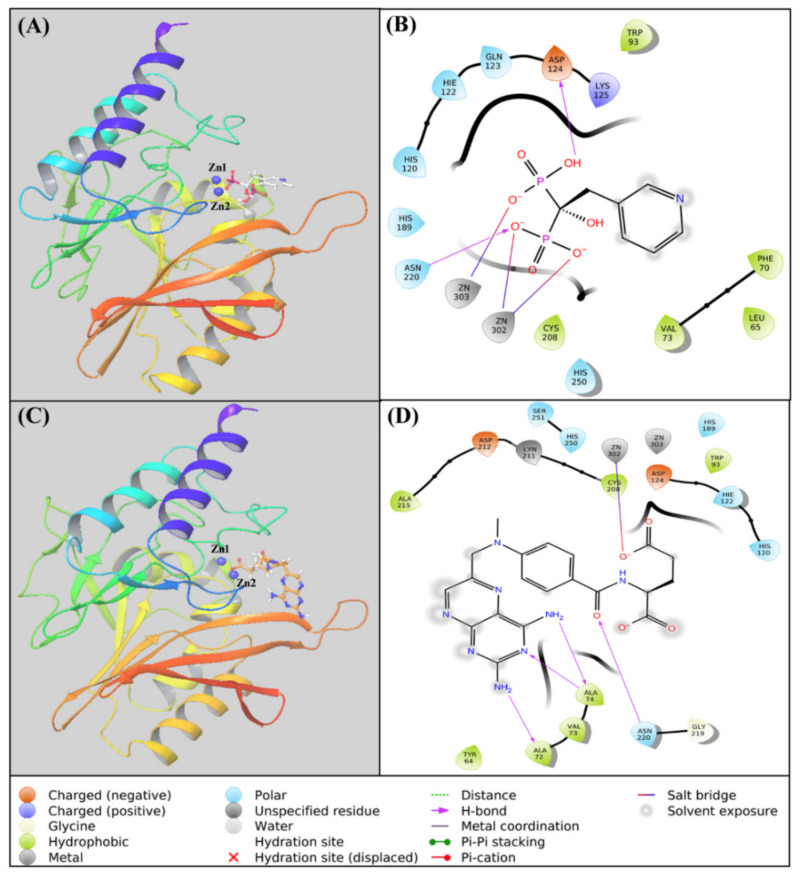
Molecular docking of NDM-1 with risedronate and methotrexate: Binding of (**A**) risedronate and (**B**) methotrexate to the active site of NDM-1. Two-dimensional representation of the interaction between (**C**) risedronate and (**D**) methotrexate.

The molecular docking between risedronate and NDM-1 revealed that it occupied the catalytic site of NDM-1 (Figure 2A). The analysis of risedronate–NDM-1 interaction suggests that the protein–drug complex was primarily stabilized by hydrogen bonding and electrostatic interactions. Risedronate formed two hydrogen bonds—with Asp124 and Asn220—while the electrostatic interactions were formed by Zn1 as well as Zn2 (two interaction) with the phosphate group of the drug (Table 3). In addition, some residues of NDM-1—such as Leu65, Phe70, Val73, Trp93, His120, His122, Gln123, Lys125, His189, Cys208, and His250—formed van der Waals interactions with risedronate (Figure 2B). The docking energy of the interaction between risedronate and NDM-1 was estimated to be −10.543 kcal mol^−1^.

#### 2.4.2. Methotrexate–NDM-1 Interaction

The molecular docking between methotrexate and NDM-1 showed that it occupied the catalytic site of NDM-1 (Figure 2C). The analysis of methotrexate–NDM-1 interaction suggests that the protein–drug complex was primarily stabilized by hydrogen bonding. Methotrexate formed four hydrogen bonds with Ala72, Ala74 (two interactions), and Asn220. An electrostatic interaction was formed between Zn1 and the drug molecule (Table 3). In addition, some residues of NDM-1—such as Zn2, Tyr64, Val73, Trp93, Asp124, His120, His122, His189, Cys208, Lys211, Asp212, Ala215, Gly219, His250, and Ser251—formed van der Waals interactions with methotrexate (Figure 2D). The docking energy of the interaction between methotrexate and NDM-1 was estimated to be −10.189 kcal mol^−1^.

### 2.5. Analysis of MD Simulation

#### 2.5.1. Root-Mean-Square Deviation (RMSD)

**Figure 3:**
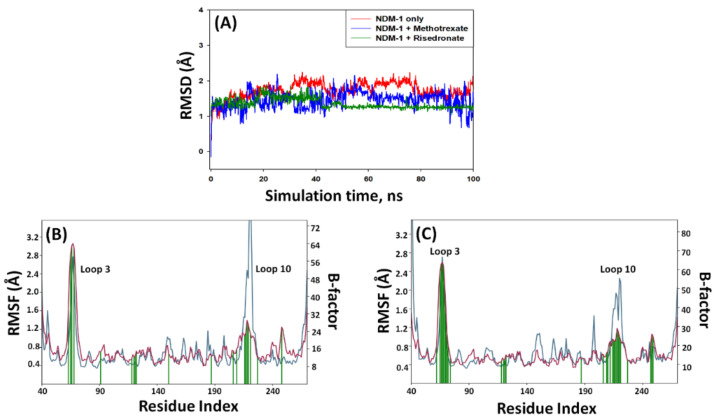
Molecular docking simulation of NDM-1 in complex with risedronate and methotrexate: (**A**) Root-mean-square deviation (RMSD) in the Cα atoms of NDM-1 in the absence and presence of risedronate and methotrexate. Root-mean-square fluctuation (RMSF) in the side chains of NDM-1 complexed with (**B**) risedronate and (**C**) methotrexate.

The RMSD is a measure of deviation in the structure of the protein–ligand complex from the initial structure during MD simulation. Figure 3A shows the RMSD values of NDM-1 alone, NDM-1–risedronate, and NDM-1–methotrexate complexes as a function of simulation time. The RMSD values were within the acceptable limit of 2.0 Å, suggesting that the protein structure did not deviate significantly during MD simulation [19]. The average RMSD of NDM-1 alone, NDM-1–risedronate, and NDM-1–methotrexate complexes was 1.84 ± 0.36 Å, 1.73 ± 0.27 Å, and 1.42 ± 0.21 Å, respectively. These results clearly indicate the formation of a stable complex between NDM-1 and drug molecules.

#### 2.5.2. Root-Mean-Square Fluctuation (RMSF)

The fluctuations in the side chain of a protein during MD simulation can be easily measured by calculating RMSF [20]. The RMSF of NDM-1–risedronate and NDM-1–methotrexate was measured and compared with the experimentally determined B-factor (during X-ray crystallography) of NDM-1 alone (Figure 3B,C). At the N- and C-terminal ends, the RMSF values were higher due to the flexible nature of the terminals. Some amino acid residues of NDM-1 showed considerably higher RMSF values, as a result of entry or binding of risedronate and methotrexate at the substrate-binding site of NDM-1. Moreover, the RMSF peaks around amino acid residues 30–40 and 170–180 were due to the higher flexibility of loop 3 and loop 10, respectively.

#### 2.5.3. Radius of Gyration (rGyr)

**Figure 4:**
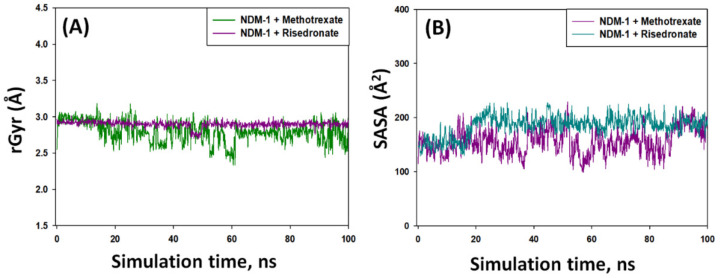
Variation in the (**A**) radius of gyration (rGyr) and (**B**) solvent accessible surface area (SASA) of NDM-1–risedronate and NDM-1–methotrexate complexes.

The binding of a ligand to a protein may affect its overall stability and folding state, which during MD simulation can be measured by observing rGyr as a function of simulation time [21]. Figure 4A shows the behavior in rGyr of NDM-1–risedronate and NDM-1–methotrexate complexes during simulation. The rGyr of NDM-1–risedronate complexes was in the range of 2.75–2.96 Å, while the rGyr of NDM-1–methotrexate complexes varied in the 2.43–3.18 Å range. Comparatively, NDM-1–methotrexate complexes showed higher fluctuations than the NDM-1–risedronate complexes; however, the fluctuations in both cases were within the acceptable limits [21]. The average rGyr values of NDM-1–risedronate and NDm-1–methotrexate complexes were 2.83 ± 0.57 Å and 2.71 ± 0.69 Å, respectively. It is clear that the overall fluctuations in the rGyr values of both complexes were not significant, suggesting stable natures of the protein–ligand complexes.

#### 2.5.4. Solvent-Accessible Surface Area (SASA)

The overall packing and stability of a protein–ligand complex during MD simulation can also be measured by estimating SASA [22]. The SASA measures the exposure of a protein–ligand complex to the surrounding solvent molecules and, therefore, measures the conformational stability of the protein–ligand complex. Figure 4B shows the behavior of SASA during MD simulation of NDM-1–risedronate and NDM-1–methotrexate complexes. We observed some minor fluctuations in the SASA of both complexes, but they remained within the acceptable limits. The SASA of NDM-1–risedronate and NDM-1–methotrexate complexes was in the range of 147–208 Å^2^ and 101–210 Å^2^, respectively. The average SASA of NDM-1–risedronate and NDM-1–methotrexate complexes was 183 ± 23 Å^2^ and 148 ± 31 Å^2^, respectively. Overall, the results of SASA along with rGyr suggested the formation of stable NDM-1–risedronate and NDM-1–methotrexate complexes.

### 2.6. Protein–Ligand Interaction Analysis

**Figure 5:**
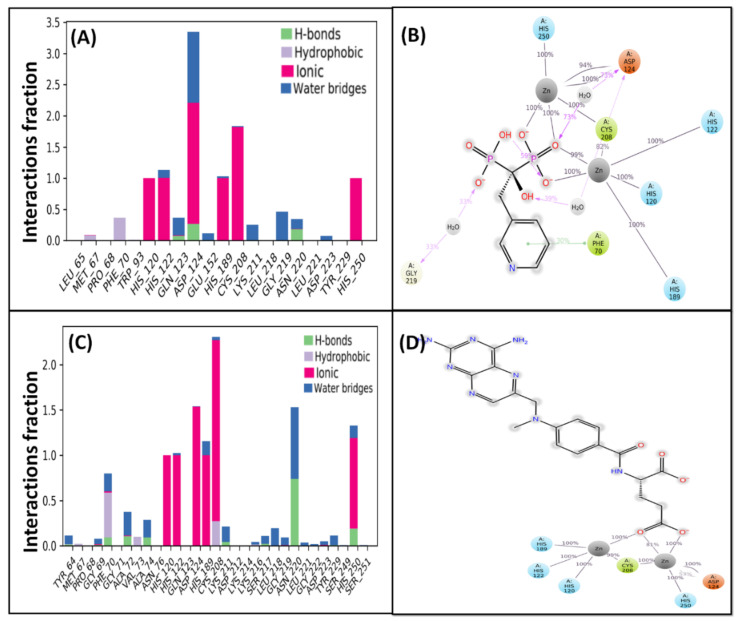
Interaction of NDM-1 with risedronate and methotrexate during MD simulation: (**A**) Interaction fraction of NDM-1 residues with risedronate, (**B**) Percentage of simulation time during which NDM-1 residues interacted with risedronate, (**C**) Interaction fraction of NDM-1 residues with methotrexate, and (**D**) Percentage of simulation time during which NDM-1 residues interacted with methotrexate.

**Figure 6:**
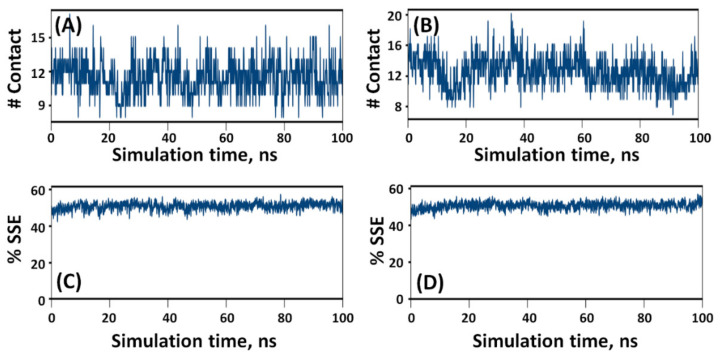
Variation in the number of contacts between NDM-1 and risedronate (**A**) and methotrexate (**B**). Variation in the secondary structure elements (SSEs) of NDM-1 in the presence of (**C**) risedronate and (**D**) methotrexate.

A preliminary examination of MD simulation of NDM-1–risedronate and NDM-1–methotrexate complexes suggested the participation of ionic interactions in stabilizing both complexes (Figure 5A,C). In addition, hydrogen bonds and hydrophobic interactions also played crucial roles in stabilizing the protein–drug complexes [20]. In NDM-1–risedronate complexes, ionic interactions were formed throughout the simulation by the active site residues His120, His122, Asp124, His189, Cys208, and His250 (Figure 5A); amongst them, Asp124 formed two interactions with risedronate through water bridges for 39% and 73% simulation time. In addition, Gly219 interacted with risedronate through a water bridge for 33%, while Phe70 formed a hydrophobic interaction with risedronate. Moreover, both of the Zn ions interacted with risedronate for almost 100% of the simulation time (Figure 5B). In NDM-1–methotrexate complexes, His120, His122, Asp124, His189, Cys208, and His250 formed ionic interactions, while Phe70, and Asn220 were involved in hydrophobic and hydrogen bond interactions, respectively, with the drug molecule (Figure 5C). Both of the Zn ions were found to interact with the carboxyl group of methotrexate for 100% of the simulation time (Figure 5D). The total numbers of contacts formed by risedronate and methotrexate with NDM-1 during MD simulation were estimated to vary from 8 to 17 and 8 to 20, respectively, suggesting a stable interaction between proteins and drugs (Figure 6A,B). Furthermore, the variation in the secondary structure elements (SSEs) of NDM-1 in the presence of risedronate and methotrexate was also monitored as a function of simulation time. The %SSE of NDM-1 in the presence of risedronate was estimated to be 50.8%, comprising 24.9% α-helices and 25.9% β-sheets. Similarly, the %SSE of NDM-1 in the presence of methotrexate was found to be 50.6%, comprising 24.9% α-helices and 25.7% β-sheets (Figure 6C,D). These results suggest that, overall, the secondary structure of NDM-1 remained consistent throughout the MD simulation.

### 2.7. Analysis of Enzyme Kinetics Parameters

The potential of the identified drugs—namely, risedronate and methotrexate—to inhibit NDM-1 activity was evaluated by performing steady-state enzyme kinetics. The NDM-1 enzyme alone was found to hydrolyze different substrates, such as ampicillin, cefotaxime, imipenem, meropenem, and the chromogenic substrate nitrocefin (Table 4). The Km, kcat, and enzyme efficiency (kcat/Km) of NDM-1 against different substrates in the absence of any inhibitor were estimated to be in the range of 23.2–96.5 µM, 242.9–758.0 s^−1^, and 3.88–10.47 µM^−1^ s^−1^, respectively (Table 4). These results are consistent with those of previously published reports [6,8,23]. However, in the presence of risedronate, the Km of NDM-1 against different substrates was increased by 1.5–2.6-fold, the kcat values decreased by 1.6–4.1-fold, and the catalytic efficiency (kcat/Km) decreased by 3.5–7.1-fold. Similarly, in the presence of methotrexate, the Km of NDM-1 against different substrates was increased by 1.2–2.5-fold, the kcat values decreased by 1.3–3.3-fold, and the catalytic efficiency (kcat/Km) decreased by 2.5–4.9-fold (Table 4). Furthermore, the enzyme kinetics parameters of NDM-1 in the presence of a known inhibitor (D-captopril) were also determined. There was no observable hydrolysis of antibiotics except for nitrocefin, when the enzyme was pre-incubated with D-captopril. The Km of NDM-1 in the presence of D-captopril using nitrocefin as a substrate was increased by 3.9-fold, while the kcat and kcat/Km values were decreased by 1.4- and 5.6-fold, respectively.

### 2.8. Analysis of IC_50_ Values

**Figure 7:**
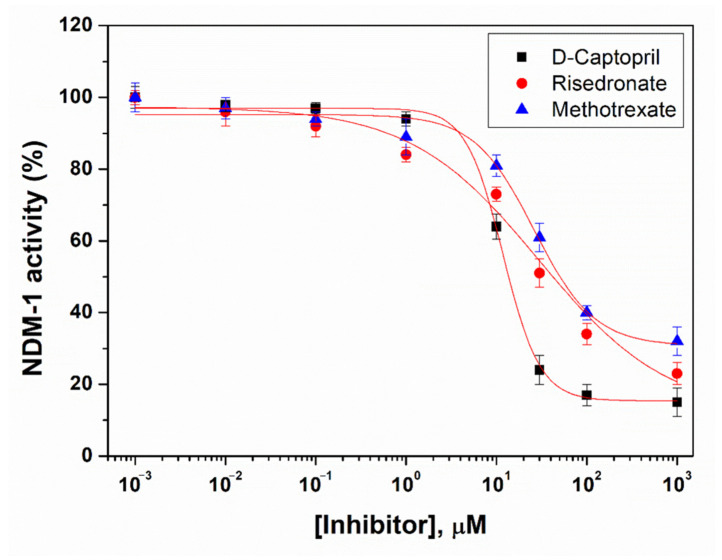
The IC_50_ curves of risedronate and methotrexate, as compared to a known inhibitor (D-captopril) of NDM-1.

The potential of risedronate and methotrexate was evaluated by determining their IC_50_ values and comparing them with that of a known NDM-1 inhibitor, i.e., D-captopril (Figure 7). The IC_50_ values of risedronate, methotrexate, and D-captopril were estimated to be 24.6 µM, 29.7 µM, and 11.8 µM, respectively [8]. Earlier, the IC_50_ value of D-captopril was reported to be 7.9–8.3 µM [8], which was close to the value obtained in this study. Since the IC_50_ values of risedronate and methotrexate were only 2.0–2.5-fold higher than that of the known inhibitor (D-captopril), this suggests the potential of the identified drug molecules as potent inhibitors of NDM-1.

### 2.9. Analysis of MIC Values

In order to evaluate the synergistic effect of risedronate and meropenem, the MICs of imipenem and meropenem were determined on an *E. coli* (ATCC BAA-2471) strain (Table 5). This strain harbors the *bla*_NDM-1_ gene, and is resistant against imipenem, meropenem, and ertapenem, while it shows sensitivity towards nitrofurantoin and is intermediate towards tigecycline. In the absence of inhibitors, MIC values of 16 mg/L were obtained for imipenem as well as meropenem. However, in the presence of risedronate, the MICs of imipenem and meropenem were reduced to 8 mg/L, while in the presence of methotrexate, the MICs of imipenem and meropenem were decreased to 4 mg/L and 2 mg/L, respectively. These results indicate that the carbapenem-resistant *E. coli* strain was re-sensitized towards imipenem and meropenem in the presence of methotrexate. Conversely, risedronate showed weaker synergy with imipenem and meropenem, as it was able to reduce the MICs by only twofold.

## 3. Discussion

The widespread dissemination of NDM-1 is a global health threat, and also poses a huge economic burden. To date, there are no specific clinical inhibitors available against NDM-1 [1,24]. This scarcity and necessity motivated us to identify novel non-β-lactam ring-containing inhibitors against NDM-1. The advantage of using non-β-lactam core-containing inhibitors is that they are not hydrolyzed and inactivated by the present defense mechanisms of the antibiotic-resistant bacteria [25]. The identification of non-β-lactam core-containing inhibitors is an optimistic way to maintain the efficacy of β-lactam antibiotics. Keeping this in mind, we adopted a multidimensional drug repurposing approach to find novel inhibitors of NDM-1 by screening an FDA-approved drugs library available at Selleck Chemicals. NDM-1 is a suitable target to overcome the problem of antibiotic resistance, as it can hydrolyze almost all available antibiotics, including carbapenems, which are considered the last resort of antibiotics [5,26,27]. Previously, NDM-1 has been used a target of choice to design/develop novel inhibitors such as ethylenediamine derivatives [28,29], pyridine derivatives [30], spiro-indole-thiadiazole derivatives [31], magnolol derivatives [32], pterostilbenes [33], sulfur-containing carboxylic acids [9,10], dithioazolidine derivatives [34], dipicolinic acids [35], phosphates [36], cyclic borates [11], Bi(III) compounds [37], chromones [38], sulfonamides [12], triazothioacetamides [39], and natural compounds [13].

In this study, we screened a library of 2685 FDA-approved drugs using high-throughput virtual screening, along with standard-precision (SP) and extra-precision (XP) molecular docking with increasingly stringent scoring functions. Finally, we identified seven drug molecules—namely, risedronate, methotrexate, pamidronate, fludarabine, alendronate, etidronate, and ibandronate—as the most potent inhibitors of NDM-1. Methotrexate is a chemotherapeutic and immune system suppressant drug, generally used to treat cancer (breast cancer, leukemia, lung cancer, gestational trophoblastic disease, osteosarcoma, etc.), autoimmune diseases (psoriasis, rheumatoid arthritis, and Crohn’s disease), and ectopic pregnancy (for medical abortion) [40]. Fludarabine is a purine analog, and is used as an antineoplastic agent for the treatment of chronic lymphocytic leukemia, non-Hodgkin’s lymphoma, acute myeloid leukemia, and acute lymphocytic leukemia [41]. Risedronate, pamidronate, alendronate, etidronate, and ibandronate are bisphosphonate drugs, generally used to treat osteoporosis and Paget’s disease of bone [42]. Bisphosphonates act by inducing apoptosis of osteoclasts, thereby inhibiting the rate of bone removal by osteoclasts [43].

Free energy calculation by MM/GBSA is a widely used parameter to ascertain the stability of a protein–ligand complex [44]. We calculated the free energy of the shortlisted drugs (risedronate, methotrexate, pamidronate, fludarabine, alendronate, etidronate, and ibandronate) to estimate the thermodynamics of drug molecules inside the binding pocket of NDM-1. Risedronate and methotrexate were found to possess the lowest MM/GBSA scores, and were therefore selected for further analysis. The stability of NDM-1–risedronate and NDM-1–methotrexate complexes was probed by MD simulation. The MD simulation results (RMSD, RMSF, rGyr, and SASA) suggest the formation of stable NDM-1–risedronate and NDM-1–methotrexate complexes. An analysis of docking poses indicated that risedronate interacted with NDM-1 through electrostatic interactions with both Zn1 and Zn2, a hydrogen bond with the catalytic residue Asp124, and van der Waals interactions with other catalytic residues such as His120, His122, His189, Cys208, and His250. Similarly, methotrexate formed an electrostatic interaction with Zn1 and van der Waals interactions with some catalytic residues, such as Asp124, His120, His122, His189, and His250. These residues play significant roles in maintaining the proper orientation of the active site and hydrolysis of β-lactam antibiotics [45]. A close analysis of NDM-1’s structure revealed that it comprises a four-layered αβ/βα fold harboring a profound and extensive active site. The two Zn ions at the active site are positioned in tetrahedral (Zn1 ion) and trigonal pyramidal (Zn2 ion) geometries. The Zn1 ion is coordinated with His120, His122, and His189, while the Zn2 ion coordinates with Asp124, Cys208, and His250 [44,45]. In addition to catalytic residues, risedronate and methotrexate interacted with some crucial non-catalytic residues such as Leu65, Trp93, and Asn220. At the time of substrate binding, Met67 reorients itself and moves away from the di-Zn center by ~4.9 Å which, in turn, brings Leu65 closer to the di-Zn center by ~2.1 Å [45]. These movements allow the substrate to enter the active site with the help of an interaction between the hydrophobic patch (Leu65, Met67, and Trp93) and the R1 group of the substrate. Moreover, as a result of active site reorientation upon substrate binding, the residue Asn220 is pulled ~1.0 Å closer to the di-Zn center and interacts with the carbonyl group of the substrate. Asn220 along with Zn1 generates an oxyanion hole at the substrate and facilitates hydrophilic attack by a hydroxide ion. The hydroxide ion is generated from the water molecule attached to Asp124 [8]. All of these results suggest that risedronate and methotrexate formed a stable complex with NDM-1 by interacting with crucial catalytic as well as non-catalytic residues.

The inhibitory potential of risedronate and methotrexate was also confirmed by enzyme kinetics. An increase in Km values suggests that both of the drug molecules occupied the active site of the enzyme, and inhibited the activity of NDM-1, as evident from a decrease in kcat values. The overall catalytic efficiency (kcat/Km) of NDM-1 in the presence of substrates/antibiotics was decreased significantly. Moreover, the IC_50_ values of risedronate and methotrexate were in the micromolar range, comparable to that of a known NDM-1 inhibitor (D-captopril). The results of molecular docking/simulation, enzyme kinetics, and IC_50_ values suggest that risedronate had a higher inhibitory potential than methotrexate. Furthermore, to confirm these observations, we determined the MICs of both drug molecules on *E. coli*. There was a twofold decrease in the MIC values of imipenem and meropenem in the presence of risedronate. However, the MIC values of imipenem and meropenem were reduced by four- and eightfold, respectively, in the presence of methotrexate. This observation was in contrast to the findings of in vitro and in silico experiments, which showed higher inhibitory potential of risedronate towards NDM-1. One possibility of a lower synergistic effect of risedronate is the presence of a negatively charged phosphate group, which may hinder the translocation of drug molecules through the lipid bilayer of Gram-negative bacteria. However, under certain environmental conditions, bacteria can express some porin channels—such as Pho E, which specifically facilitates the transfer of anionic molecules through the lipid bilayer [46]. Furthermore, this hindrance can be overlooked by using liposome-coated delivery vehicles for the delivery of risedronate into the bacterial cells [47]. Previously, chitosan-coated liposomes containing risedronate have been reported for the delivery of drugs for better absorption in the gastrointestinal tract [48].

Despite the NDM-1-inhibitory potential of methotrexate, it should be noted that its high dose (>500 mg/m^2^, which is often administered during cancer treatment) is known for toxicity [49]. Some of the reasons for the development of methotrexate toxicity include increased patient susceptibility while undergoing treatment, administration of excessive dosage by error, etc. Moreover, methotrexate is also sometimes used by individuals themselves without prescription in order to abort pregnancies, which may lead to drug overdose. Methotrexate toxicity may last from hours to days or even weeks; however, there are several ways available to overcome methotrexate toxicity. One such example includes the administration of activated charcoal if there has been a recent oral overdose of the drug. In the case of renal toxicity, adequate hydration and urinary alkylation may reduce the toxicity. More importantly, medications such as leucovorin, thymidine, and glucarpidase can be used as antidotes to methotrexate toxicity. Glucarpidase in combination with leucovorin has been recently used as antidote to methotrexate toxicity; it acts by converting methotrexate to an inactive form (DAMPA: 4-deoxy-4-amino-*N*-10-methylpteroic acid), and quickly lowers the drug’s level in the blood [49]. Therefore, before approving methotrexate as an NDM-1 inhibitor, its dosage and potential toxicity should be addressed.

In sum, risedronate and methotrexate bind to the active site of NDM-1 and form a stable complex. As the binding potential of these compounds towards NDM-1 is higher than that of β-lactam antibiotics, they compete with or replace antibiotics at the enzyme’s active site, enabling the latter to survive.

## 4. Materials and Methods

### 4.1. Materials

The antibiotics—namely, ampicillin, cefotaxime, imipenem, and meropenem—and PAR (4-(2-pyridylazoresorcinaol) were procured from Sigma (St. Louis, MO, USA). Nitrocefin was bought from Calbiochem (St. Louis, MO, USA). All of the reagents and chemicals were of analytical grade. The inhibitors—i.e., risedronate, methotrexate, and D-captopril—were purchased from Mcule Inc. (Palo Alto, CA, USA).

### 4.2. Preparation of Ligands and Protein

The library of FDA-approved drugs (L1300) was downloaded from Selleck Chemicals (Pittsburgh, PA, USA) from https://www.selleckchem.com/screening/fda-approved-drug-library.html (accessed on 2 October 2020). The 2685 drugs present in the library were subjected to HTVS (high-throughput virtual screening) against NDM-1 using Glide (Schrodinger, LLC, New York, NY, USA), as described previously [50]. Prior to HTVS, the ligands were prepared by assigning ionization states at pH 7.5 ± 2.0, followed by salt removal using the Epik function of the LigPrep module (Schrodinger, LLC, New York, NY, USA). For each ligand, a maximum of 32 conformations were generated and their energies were minimized using an OPLS3a (optimized potentials for liquid simulations) force field, as reported earlier [51].

The three-dimensional coordinates of NDM-1 were retrieved from the RCSB databank (https://www.rcsb.org/structure/4EYL) (accessed on 2 October 2020). The X-ray crystal structure of NDM-1 bound to hydrolyzed methicillin (PDB ID: 4EYL) was resolved to 1.90 Å [45]. For molecular docking, the Protein Preparation Wizard (Glide, Schrodinger, LLC, New York, NY, USA) was employed to optimize the structure of NDM-1 by adding H atoms, assigning bond orders, and removing any heteroatoms—including non-essential (non-catalytic) water molecules. The changes on Zn ions were maintained. Any missing loops or side chains of residues were added using Prime (Schrodinger, LLC, New York, NY, USA). A network of hydrogen bonds was created before minimizing the energy of the whole system using the OPLS3a force field. Molecular docking was performed inside a grid box of 27 Å × 27 Å × 27 Å dimensions, located at 2.4 Å × −40.8 Å × 1.8 Å. The grid box was defined by picking the bound ligand—i.e., meropenem in the crystal structure of NDM-1—as the centroid of the grid box in Maestro (Schrodinger, LLC, New York, NY, USA).

### 4.3. Validation of Docking Protocol

The authenticity of the docking procedure adopted in this study was confirmed by extracting the ligand (i.e., methicillin) from the X-ray crystal structure of NDM-1 and re-docking again at the active site. Finally, the docked pose and the crystal structure pose of the ligand were superimposed, and the RMSD (root-mean-square deviation) between the two poses was calculated. Furthermore, the enrichment calculator in Maestro (Schrodinger, LLC, New York, NY, USA) was utilized to validate the ability of the adopted docking protocol to predict the active compounds from a pool of inactive decoys, as well as whether it could predict the active ligands in the top percentiles of a ranked database [52]. The Schrodinger-based decoys were employed for enrichment purposes [53]. The ligands in the decoy database were processed and docked into the binding cavity of NDM-1, as described in Section 4.2. As a post-docking filter, the ligands not interacting with the catalytic residues were ignored. The efficacy of the docking protocol was evaluated by AUCROC curves. The AUC and BEDROC values were determined at 1 and 20%, respectively.

### 4.4. Molecular Docking

Molecular docking of FDA-approved drugs (L1300, Selleck Inc., Pittsburgh, PA, USA) against NDM-1 was performed in three stages using Glide (Schrodinger, LLC, New York, NY, USA), as described previously [54,55]. In the first stage, HTVS was conducted on the FDA-approved drugs to get a rough estimation of the docking energies of ligands towards the target protein. In the second stage, the ligands displaying good docking energies in HTVS were further subjected to SP docking, wherein the protein–ligand complexes were optimized by post-docking minimization. In the third stage, the ligands shortlisted after SP docking were subjected to more stringent XP docking, wherein the scaling factor and partial charge cutoff were set to 0.80 and 0.15, respectively [55]. Finally, the ligands showing the best docking energies towards NDM-1 were subjected to post-docking analysis in Maestro (Schrodinger, LLC, New York, NY, USA).

### 4.5. Free Energy Calculation by MM/GBSA (Molecular Mechanics/Generalized Born Surface Area)

The free energies for the interaction between NDM-1 and the shortlisted drug molecules were calculated by molecular mechanics force fields and the implicit solvation method using Prime (Schrodinger, LLC, New York, NY, USA), as reported previously [56,57]. In this method, the energies of docked poses were first minimized using the local optimization feature of the Prime module, and then the binding energies were computed via an MM/GBSA continuum solvation protocol utilizing an OPLS3a force field, a VSGB solvation model, and a rotamer search algorithm [51]. The free energy was calculated using the following relationship:

$$\Delta G=G_{complex\_minimized}-\left[G_{ligand\_minimized}+G_{protein\_minimized}\right]$$

where *G_Complex_minimized_*, *G_Ligand_minimized_*, and *G_Protein_minimized_* were the minimized free energies of the protein–ligand complex, ligand only, and protein only, respectively.

### 4.6. Molecular Dynamics (MD) Simulation

MD simulation of NDM-1 and drug complexes was performed using Desmond (Schrodinger, LLC, New York, NY, USA) to evaluate their stability and dynamics, as described previously [58,59,60]. A 100 ns MD simulation was performed inside an orthorhombic box after placing the NDM-1 and drug complex at the center of the box in such a way that the boundaries of the box were at least 10 Å away. The box was solvated with a TIP3P explicit water model and neutralized by adding the proper Na^+^ and Cl^−^ ions. The physiological conditions were mimicked by adding 150 mM NaCl. The Zn ions were modeled using a non-bonded model. A total of 2000 iterations were performed in order to minimize the system, keeping a convergence criterion of 1 kcal/mol/Å. The MD simulation was performed at 300 K temperature and 1.013 bar pressure (NTP ensemble), with default relaxation settings. The temperature and pressure were maintained using a Nose–Hoover chain thermostat and a Martyna–Tobias–Klein barostat, respectively [61,62]. The time step was set at 2 fs. The energy and structure were logged every 10 ns and stored in the trajectory file.

### 4.7. Determination of Enzyme Kinetics Parameters

The NDM-1 enzyme was purchased from GenScript (Piscataway, NJ, USA), and its purity was confirmed by SDS–PAGE. The zinc content of the enzyme was accessed using PAR (4-(2-pyridylazo) resorcinol) assay, as described previously [6]. A molar extinction coefficient of 27,800 M^−1^ cm^−1^ was used to determine the concentration of NDM-1 via a spectrophotometer. Furthermore, the potential of shortlisted drug molecules to inhibit the hydrolytic activity of NDM-1 was determined by steady-state enzyme kinetics, as reported previously [8]. The hydrolysis of nitrocefin (synthetic substrate), ampicillin, cefotaxime, imipenem, and meropenem was monitored in the absence and presence of drug molecules. The changes in the molar extinction coefficients of the chosen antibiotics/substrates were Δε_486_ = +15,000 M^−1^ cm^−1^ for nitrocefin, Δε_235_ = −900 M^−1^ cm^−1^ for ampicillin, Δε_264_ = −7250 M^−1^ cm^−1^ for cefotaxime, Δε_295_ = −10,500 M^−1^ cm^−1^ for imipenem, and Δε_297_ = −10,940 M^−1^ cm^−1^ for meropenem. The reaction was carried out in 50 mM HEPES buffer (pH 7.0) containing 250 mM NaCl, and 100 µM ZnCl_2_ at 30 °C. To prevent denaturation of NDM-1, 20 µg/mL BSA (no hydrolytic activity of its own) was added to the reaction buffer. The concentrations of different substrates/antibiotics—namely, nitrocefin, ampicillin, cefotaxime, imipenem, and meropenem—were in the ranges of 0–150 µM, 0–600 µM, 0–300 µM, 0–400 µM, and 0–250 µM, respectively. The concentrations of risedronate and methotrexate were kept at 25 µM and 30 µM, respectively. The initial velocities were calculated from the observed hydrolysis and the kinetic parameters—namely, Km and kcat were determined using the following Michaelis–Menten equations:

$$v=\frac{V_{max}\;\left[S\right]}{K_{m}+\left[S\right]}$$

$$k_{cat}=\frac{V_{max}}{\left[E\right]}$$

where *v*, *V*_max_, [*S*], and [*E*] are initial velocity, maximum velocity, substrate concentration, and enzyme concentration, respectively.

### 4.8. Evaluation of IC_50_ Values

The IC_50_ of an inhibitor is the concentration at which it inhibits the enzyme activity by 50%. The IC_50_ values of risedronate and methotrexate, along the control inhibitor (D-captopril), were evaluated by using nitrocefin as substrate. Different concentrations of inhibitors (0.001 to 1000 µM) were incubated with NDM-1 at 30 °C for 5 min, and the activity of NDM-1 was determined by monitoring the hydrolysis of nitrocefin (100 µM).

### 4.9. Determination of Minimum Inhibitory Concentration (MIC)

The MICs of imipenem meropenem in the presence or absence of risedronate and methotrexate were determined using *E. coli* (ATCC-BAA-2471) via microbroth dilution method, and the results were interpreted according to EUCAST (European Committee on Antimicrobial Susceptibility Testing) [63]. The concentrations of antibiotics (imipenem and meropenem) were in the range of 0.0625 to 64 mg/L in double dilutions, while the concentrations of risedronate and methotrexate were kept constant at 32 mg/L. An inoculum of ~5 × 10^5^ CFU/mL was used to grow *E. coli* in cation-adjusted Mueller–Hinton broth at 37 °C for 20 ± 2 h. MIC is defined as the lowest concentration at which no visible growth of bacteria was observed.

## 5. Conclusions

The drug-repurposing approach is a rapid strategy to identify and develop potential inhibitors against β-lactamases. In this study, an FDA-approved drug library was screened first by high-throughput virtual screening, and then by standard-precision and extra-precision molecular docking approaches. On the basis of the docking score, seven drug molecules (risedronate, methotrexate, pamidronate, fludarabine, alendronate, etidronate, and ibandronate) were found to occupy the active site of NDM-1, with high affinity. Amongst the shortlisted drug molecules, risedronate and methotrexate showed the lowest free energy as calculated by MM/GBSA. An analysis of molecular docking poses confirmed that these drugs occupied the substrate-binding site of NDM-1. The Zn ions of NDM-1 also interacted electrostatically with risedronate as well as methotrexate. The catalytic amino acid residues—such as His120, Asp124, His122, His189, Cys208, and His250—interacted with risedronate and methotrexate mainly through hydrogen bonds and hydrophobic interactions. In addition, some non-active site residues (e.g., Phe70, Val73, Trp93, and Asn220) that play crucial role in the hydrolysis of β-lactam antibiotics were also involved in stabilizing NDM-1–drug interaction. Furthermore, the stability of NDM-1–risedronate and NDM-1–methotrexate complexes was evaluated by molecular dynamics (MD) simulation. The potential of risedronate and methotrexate to inhibit NDM-1 activity was confirmed by in vitro enzyme kinetics and MIC determination. Hence, risedronate and methotrexate may serve as lead scaffolds in the further design and optimization of inhibitors against β-lactamases.

## Figures and Tables

**Table 1:** Extra precision (XP) docking parameters of drugs shortlisted on the basis of SP score (≤ −8.0 kcal mol^−1^).

S. No.	Name of Drug	Docking Score (kcal mol^−1^)	Glide g-Score (kcal mol^−1^)	Glide e-Model (kcal mol^−1^)
1.	Risedronate	−10.355	−10.543	−63.790
2.	Methotrexate	−10.124	−10.189	−71.625
3.	Pamidronate	−9.955	−10.066	−49.713
4.	Fludarabine	−9.952	−10.044	−64.162
5.	Alendronate	−9.899	−10.098	−47.836
6.	Etidronate	−9.764	−10.01	−57.003
7.	Ibandronate	−9.297	−9.388	−54.462
8.	Raltitrexed	−8.713	−8.777	−80.917
9.	Tenofovir	−8.332	−8.913	−51.885
10.	Mizoribine	−7.915	−7.928	−54.449
11.	Carbenicillin	−7.895	−7.895	−58.606
12.	Ticarcillin	−7.809	−7.809	−54.033
13.	Foscarnet	−7.554	−7.561	−38.003
14.	Procodazole	−7.099	−7.141	−45.689
15.	Pasiniazid	−7.083	−7.084	−35.139
16.	Nalidixic acid	−7.012	−7.057	−41.417
17.	Zalcitabine	−6.859	−6.859	−50.197
18.	Sodium butyrate	−6.623	−6.627	−26.326
19.	Acipimox	−6.471	−6.474	−33.279
20.	Epalrestat	−6.364	−6.364	−45.096
21.	Carboplatin	−6.176	−7.200	−40.200
22.	Ethamsylate	−6.153	−6.154	−39.315
23.	Gimeracil	−5.329	−5.329	−39.587

**Table 2:** Molecular mechanics/generalized Born surface area (MM/GBSA) of drugs showing lower docking energies (≤−8.0 kcal mol^−1^) in XP docking.

Name of Drug	Δ*G*_*Bind*_	Δ*G*_*Coulomb*_	Δ*G*_*Covalent*_	Δ*G*_*H-Bond*_	Δ*G*_*Lipo*_	Δ*G*_*Packing*_	Δ*G*_*Solv-GB*_	Δ*G*_*vdW*_
Risedronate	−36.51	−85.12	4.46	−1.88	−5.66	−0.65	82.97	−30.63
Methotrexate	−44.16	−94.41	8.32	−3.44	−6.41	−1.51	89.80	−36.50
Pamidronate	−20.93	−80.09	4.93	−1.90	−0.96	0	75.54	−18.45
Fludarabine	−19.25	−96.01	6.38	−1.46	−4.24	−3.01	104.21	−25.13
Alendronate	−22.52	−82.80	4.14	−1.86	−1.75	0	77.67	−17.90
Etidronate	−20.78	−94.83	4.59	−1.89	−0.49	0	86.95	−15.10
Ibandronate	−24.53	−95.89	7.36	−2.64	−8.53	0	99.19	−24.03

**Table 3:** Docking parameters for the interaction between identified inhibitors and NDM-1.

Name of Drug	Hydrogen Bonding	Electrostatic Interaction	Van Der Waals Interactions	XP Docking Score, Δ*G* (kcal mol^−1^)
Risedronate	**Asp124**, Asn220	Zn1, Zn2 *	Leu65, Phe70, Val73, Trp93, **His120**, **His122**, Gln123, Lys125, **His189**, **Cys208**, **His250**	−10.543
Methotrexate	Ala72, Ala74 ^*^, Asn220	**Zn1**	**Zn2**, Tyr64, Val73, Trp93, **Asp124**, **His120**, **His122**, **His189**, **Cys208**, Lys211, Asp212, Ala215, Gly219, **His250**, Ser251	−10.189

* Indicates two interactions. Residues in bold are catalytic active site residues of NDM-1.

**Table 4:** Steady-state enzyme kinetics of NDM-1 in the presence of risedronate and methotrexate.

Substrates	*K*_m_ (μM)	V_max_ (μM s^−1^)	*k_cat_* (s^−1^)	*k_cat_*/*K*_m_ (μM^−1^ s^−1^)
NDM-1				
Nitrocefin	23.2 ± 1.7	0.486 ± 0.026	242.9 ± 15.2	10.47 ± 1.01
Ampicillin	96.5 ± 3.1	0.749 ± 0.045	374.7 ± 14.3	3.88 ± 0.19
Cefotaxime	54.8 ± 3.7	0.751 ± 0.034	375.4 ± 17.5	6.85 ± 0.56
Imipenem	75.9 ± 2.9	1.516 ± 0.068	758.0 ± 18.6	9.99 ± 0.45
Meropenem	52.8 ± 3.4	0.644 ± 0.044	322.2 ± 11.7	6.10 ± 0.45
NDM-1 + Risedronate				
Nitrocefin	60.6 ± 2.3	0.305 ± 0.023	152.7 ± 9.6	2.52 ± 0.18
Ampicillin	163.9 ± 5.1	0.364 ± 0.027	182.1 ± 8.2	1.11 ± 0.06
Cefotaxime	95.1 ± 4.5	0.182 ± 0.011	91.1 ± 3.1	0.96 ± 0.06
Imipenem	114.9 ± 7.7	0.474 ± 0.034	236.9 ± 7.9	2.06 ± 0.15
Meropenem	107.3 ± 6.2	0.232 ± 0.019	116.2 ± 5.7	1.08 ± 0.08
NDM-1 + Methotrexate				
Nitrocefin	57.9 ± 2.8	0.363 ± 0.026	181.3 ± 8.9	3.13 ± 0.22
Ampicillin	134.8 ± 7.4	0.415 ± 0.035	207.7 ± 6.1	1.54 ± 0.10
Cefotaxime	81.9 ± 6.2	0.226 ± 0.018	113.2 ± 5.4	1.38 ± 0.12
Imipenem	92.4 ± 3.6	0.557 ± 0.033	278.4 ± 7.2	3.01 ± 0.14
Meropenem	89.1 ± 5.3	0.261 ± 0.020	130.6 ± 6.8	1.47 ± 0.12
NDM-1 + D-Captopril (control)				
Nitrocefin	91.2 ± 5.6	0.214 ± 0.019	171.3 ± 4.4	1.88 ± 0.13

**Table 5:** MICs of an *E. coli* (ATCC-BAA-2469) strain carrying the *bla*_NDM-1_ gene towards imipenem and meropenem in the presence of risedronate and methotrexate.

Minimum Inhibitory Concentration (MIC) in mg/L		
	Imipenem	Meropenem
*E. coli* only	16	16
*E. coli* + Risedronate (32 mg/L)	8	8
*E. coli* + Methotrexate (32 mg/L)	4	2

## Data Availability

Not applicable.

## Supplementary Materials

The following are available online, Table S1: high-throughput virtual screening (HTVS) parameters of the FDA-approved drugs; Table S2: standard-precision (SP) docking parameters of drugs shortlisted on the basis of HTVS score (≤−7.0 kcal mol^−1^).
