# Supplementary material for: Risedronate and Methotrexate Are High-Affinity Inhibitors of New Delhi Metallo-β-Lactamase-1 (NDM-1): A Drug Repurposing Approach
Source: Molecules. 2022 Feb 14;27(4):1283. doi: 10.3390/molecules27041283 (PMC8878330; doi:10.3390/molecules27041283)
Supplement: Supplementary file 1 [file molecules-27-01283-s001.zip › molecules-1570835-supplementary.pdf]

Supplementary Materials

Risedronate and Methotrexate are High-Affinity Inhibitors of New Delhi Metallo-β-Lactamase-1 (NDM-1): A Drug Repurposing Approach

Ghazala Muteeb [1], Abdulrahman Alsultan [2], Mohd Farhan [3] and Mohammad Aatif [4,*]

[1] Department of Nursing, College of Applied Medical Science, King Faisal University, Al-Ahsa 31982, Saudi Arabia; graza@kfu.edu.sa
[2] College of Applied Medical Science, King Faisal University, Al-Ahsa 31982, Saudi Arabia; aalsultan@kfu.edu.sa
[3] Department of Basic Sciences, , King Faisal University, Al-Ahsa 31982, Saudi Arabia; mfarhan@kfu.edu.sa
[4] Department of Public Health, College of Applied Medical Science, King Faisal University, Al-Ahsa 31982, Saudi Arabia
* Correspondence: maahmad@kfu.edu.sa

Table S1. High Throughput Virtual Screening (HTVS) parameters of the FDA-approved drugs.

S. No.	Name of drugs	Docking score (kcal mol^{-1})	S. No.	Name of drugs	Docking score (kcal mol^{-1})
1.	Foscarnet	-9.380	414.	Sulfadoxine	-5.625
2.	Etidronate	-8.968	415.	(+,-)-Octopamine	-5.623
3.	Ethamsylate	-8.922	416.	L-Arginine	-5.623
4.	Risedronate	-8.808	417.	Trilostane	-5.622
5.	Pamidronate	-8.763	418.	Carbamyl-β-methylcholine	-5.616
6.	Taurine	-8.638	419.	Ornidazole	-5.612
7.	Pasiniazid	-8.524	420.	Ethinyl estradiol	-5.607
8.	Ticarcillin	-8.442	421.	Menadione	-5.606
9.	Fosbretabulin	-8.414	422.	Levodropropizine	-5.603
10.	Carboplatin	-8.353	423.	Flubendazole	-5.597
11.	Acipimox	-8.276	424.	Spectinomycin	-5.594
12.	Tenofovir	-8.245	425.	Prucalopride	-5.580
13.	Fludarabine	-8.231	426.	Moguisteine	-5.576
14.	Nateglinide	-8.230	427.	Dasatinib	-5.560
15.	Rebamipide	-8.226	428.	Galardin/Ilomastat	-5.552
16.	Tasisulam	-8.222	429.	Felodipine	-5.551
17.	Pranoprofen	-8.191	430.	Milrinone	-5.538
18.	Alendronate	-8.181	431.	Secnidazole	-5.538
19.	Azlocillin	-8.105	432.	Capecitabine	-5.528
20.	Orotic acid	-8.083	433.	Donepezil	-5.517
21.	Methotrexate	-8.065	434.	Riluzole	-5.517
22.	Mesna	-7.988	435.	Nilvadipine	-5.508
23.	Olsalazine	-7.968	436.	Rimonabant	-5.495
24.	Sulbactam	-7.962	437.	Tetramisole	-5.495

25.	Aspirin	-7.924	438.	Propylthiouracil	-5.494
26.	Nalidixic acid	-7.921	439.	Solifenacin succinate	-5.494
27.	Pircetam	-7.917	440.	Nitrendipine	-5.493
28.	Ibandronate	-7.902	441.	Chlorpropamide	-5.491
29.	Biotin	-7.866	442.	Avanafil	-5.459
30.	Carprofen	-7.851	443.	Trichlormethiazide	-5.459
31.	Sodium butyrate	-7.839	444.	Sulfadiazine	-5.459
32.	Benzoic acid	-7.828	445.	Mycophenolate mofetil	-5.458
33.	Sodium ascorbate	-7.775	446.	Thiabendazole	-5.457
34.	Ketoprofen	-7.767	447.	Ampiroxicam	-5.449
35.	Cinchophen	-7.748	448.	Tazarotene	-5.448
36.	Inosine	-7.746	449.	Raltegravir	-5.448
37.	Tranexamic acid	-7.725	450.	Benazepril	-5.447
38.	Raltitrexed	-7.716	451.	Zidovudine	-5.445
39.	Epalrestat	-7.708	452.	Terfenadine	-5.444
40.	Niacin	-7.704	453.	Etomidate	-5.441
41.	Phthalylsulfacetamide	-7.656	454.	Levamisole	-5.441
42.	Meropenem	-7.640	455.	Progesterone	-5.440
43.	Cinoxacin	-7.629	456.	Sulfaguanidine	-5.430
44.	Flumequine	-7.629	457.	Acyclovir	-5.427
45.	Tolcapone	-7.622	458.	Linagliptin	-5.409
46.	Carbenicillin	-7.611	459.	Minocycline	-5.405
47.	Procodazole	-7.611	460.	S-Ruxolitinib	-5.403
48.	Ciprofibrate	-7.604	461.	Amisulpride	-5.401
49.	Zileuton	-7.603	462.	Climbazole	-5.400
50.	Sodium picosulfate	-7.601	463.	Carvedilol	-5.392
51.	Zaltoprofen	-7.574	464.	Azelastine	-5.390
52.	Flufenamic acid	-7.568	465.	Topiramate	-5.388
53.	Methyldopa	-7.544	466.	Nabumetone	-5.377
54.	Aicar	-7.539	467.	Amfebutamone	-5.374
55.	Flucytosine	-7.539	468.	Amonafide	-5.373
56.	Pidotimod	-7.526	469.	Pemirolast	-5.373
57.	Zalcitabine	-7.525	470.	Fluocinonide	-5.370
58.	Ibuprofen	-7.514	471.	Camostat mesilate	-5.361
59.	Entecavir	-7.499	472.	Bifonazole	-5.351
60.	Chromocarb	-7.496	473.	Roxatidine	-5.344
61.	Sodium gluconate	-7.491	474.	Tebipenem pivoxil	-5.344
62.	Mizoribine	-7.481	475.	Naloxone	-5.337
63.	Triflusal	-7.480	476.	Valaciclovir	-5.332
64.	Enalaprilat	-7.471	477.	Icotinib	-5.321
65.	Oxiracetam	-7.448	478.	Palonosetron	-5.320
66.	Cytarabine	-7.438	479.	Prednisolone	-5.313
67.	Lonidamine	-7.404	480.	Levosulpiride	-5.311
68.	Cidofovir	-7.397	481.	Troxipide	-5.311
69.	Clofibric acid	-7.391	482.	Yohimbine	-5.311
70.	Diclazuril	-7.386	483.	Vinpocetine	-5.309

71.	Enoxacin	-7.372	484.	Histamine	-5.308
72.	Oxaprozin	-7.370	485.	Desonide	-5.305
73.	Sodium phenylbutyrate	-7.367	486.	Hexestrol	-5.300
74.	Etodolac	-7.355	487.	Nalmefene	-5.300
75.	Oxfendazole	-7.354	488.	Phenytoin	-5.296
76.	Chloroxine	-7.349	489.	Ractopamine	-5.296
77.	Clinofbrate	-7.345	490.	Topotecan	-5.294
78.	Furosemide	-7.341	491.	Methazolamide	-5.285
79.	Ribavirin	-7.335	492.	Loratadine	-5.284
80.	Noradrenaline	-7.328	493.	Desvenlafaxine	-5.276
81.	Piromidic acid	-7.324	494.	Apixaban	-5.275
82.	Isosorbide	-7.322	495.	Butylscopolamine	-5.269
83.	Ciclopirox	-7.311	496.	Diperodon	-5.264
84.	Adenosine	-7.291	497.	Nifedipine	-5.261
85.	Dopamine	-7.267	498.	Imiquimod	-5.257
86.	Naproxen	-7.266	499.	Lubiprostone	-5.256
87.	Suprofen	-7.263	500.	Buflomedil	-5.244
88.	Dyphylline	-7.261	501.	Candesartan	-5.244
89.	Streptozocin	-7.261	502.	Rufinamide	-5.244
90.	Olopatadine	-7.246	503.	Epinastine	-5.243
91.	Dihydroartemisinin	-7.245	504.	Prasugrel	-5.235
92.	Fluvastatin	-7.234	505.	Estradiol cypionate	-5.229
93.	Gimeracil	-7.234	506.	Moxifloxacin	-5.222
94.	Omeprazole	-7.224	507.	Metaraminol	-5.202
95.	DL-Carnitine	-7.223	508.	Meticrane	-5.198
96.	Tolfenamic	-7.195	509.	Azaperone	-5.193
97.	Niflumic acid	-7.193	510.	Levonorgestrel	-5.192
98.	Enrofloxacin	-7.172	511.	Fudosteine	-5.184
99.	Uridine	-7.172	512.	Mestranol	-5.182
100.	Felbamate	-7.167	513.	Gestodene	-5.180
101.	Nitrofurazone	-7.163	514.	Phentolamine mesilate	-5.179
102.	Mefenamic acid	-7.163	515.	Voriconazole	-5.167
103.	(R)-Baclofen	-7.158	516.	Indapamide	-5.166
104.	Diclofenac	-7.153	517.	Pilocarpine	-5.156
105.	Tranilast	-7.152	518.	Diethylstilbestrol	-5.148
106.	Isoniazid	-7.151	519.	Tinidazole	-5.147
107.	Pefloxacin mesylate	-7.133	520.	Varenicline	-5.133
108.	Febuxostat	-7.132	521.	Acebutolol	-5.131
109.	Chlorothiazide	-7.131	522.	Flavoxate	-5.124
110.	Dimesna	-7.131	523.	Nicotine	-5.124
111.	Doxifluridine	-7.115	524.	Nialamide	-5.111
112.	Sarafloxacin	-7.109	525.	Dimethyl	-5.110
113.	Glafenine	-7.105	526.	Ketotifen	-5.103
114.	Valganciclovir	-7.104	527.	Naltrexone	-5.100
115.	Bindarit	-7.103	528.	Fenofibrate	-5.084
116.	Lisinopril	-7.103	529.	Trimethoprim	-5.076

117.	Bezafibrate	-7.078	530.	Deoxycorticosterone	-5.074
118.	Indomethacin	-7.076	531.	Monobenzone	-5.062
119.	Sulindac	-7.069	532.	Spironolactone	-5.061
120.	Erdosteine	-7.065	533.	Acetanilide	-5.041
121.	Flopropione	-7.056	534.	Detomidine	-5.017
122.	L-Carnitine	-7.036	535.	Carbazochrome	-5.014
123.	Flunixin meglumine	-7.028	536.	Tioconazole	-5.013
124.	Captopril	-7.023	537.	Phenytoin	-5.009
125.	Tolmetin	-7.014	538.	Nimesulide	-5.007
126.	Chlorquinaldol	-6.998	539.	Paroxetine	-5.004
127.	Isoetharine mesylate	-6.986	540.	Aripiprazole	-4.993
128.	Probenecid	-6.979	541.	Haloperidol	-4.978
129.	Levofloxacin	-6.973	542.	Quinapril	-4.961
130.	Linezolid	-6.964	543.	Glipizide	-4.958
131.	Temozolomide	-6.957	544.	Escitalopram	-4.953
132.	Adrenalone	-6.952	545.	Urapidil	-4.952
133.	Genistein	-6.950	546.	Dienogest	-4.947
134.	Nadifloxacin	-6.950	547.	Oxybutynin	-4.947
135.	Canrenoic acid	-6.946	548.	Luliconazole	-4.944
136.	Norfloxacin	-6.946	549.	Busulfan	-4.940
137.	Acitretin	-6.934	550.	Tropisetron	-4.934
138.	Famotidine	-6.929	551.	Deflazacort	-4.926
139.	Lomefloxacin	-6.929	552.	Cleviprex	-4.917
140.	Balofloxacin	-6.928	553.	Enoxolone	-4.916
141.	Fluorouracil	-6.920	554.	Pyridostigmine	-4.912
142.	Mozavaptan	-6.919	555.	Sorafenib	-4.903
143.	Uracil	-6.915	556.	Venlafaxine	-4.898
144.	Nicotinamide	-6.913	557.	Pimozide	-4.884
145.	(S)-(+)-Flurbiprofen	-6.911	558.	Metolazone	-4.874
146.	Isoprenaline	-6.902	559.	Nisoldipine	-4.869
147.	Gabapentin	-6.891	560.	Berberine	-4.866
148.	Ellagic acid	-6.877	561.	Benzthiazide	-4.863
149.	Epinephrine	-6.872	562.	Econazole	-4.861
150.	L-Adrenaline	-6.872	563.	Altrenogest	-4.857
151.	Carbamazepine	-6.866	564.	Acetylcysteine	-4.855
152.	Glutamine	-6.866	565.	Pelitinib	-4.816
153.	Tamibarotene	-6.866	566.	Cyproterone	-4.802
154.	Levetiracetam	-6.863	567.	Ondansetron	-4.800
155.	Cyclandelate	-6.857	568.	Rizatriptan	-4.796
156.	Allopurinol	-6.847	569.	Iloperidone	-4.795
157.	Ursodiol	-6.844	570.	Procarbazine	-4.791
158.	Zoxazolamine	-6.843	571.	Doxercalciferol	-4.790
159.	Perindopril erbumine	-6.837	572.	Mevastatin	-4.780
160.	Isoxicam	-6.837	573.	Betaxolol	-4.762
161.	Emtricitabine	-6.829	574.	Medroxyprogesterone	-4.759
162.	Hydrocortisone	-6.827	575.	Clofoctol	-4.742

163.	Nelarabine	-6.823	576.	Anastrozole	-4.741
164.	Thalidomide	-6.820	577.	Sulfamethoxypyridazine	-4.732
165.	Bexarotene	-6.816	578.	Trometamol	-4.729
166.	Flupirtine	-6.816	579.	Rivastigmine	-4.720
167.	Cladribine	-6.800	580.	Agomelatine	-4.718
168.	Prednisone	-6.788	581.	Drospirenone	-4.718
169.	Phenindione	-6.780	582.	Famciclovir	-4.711
170.	Ciclesonide	-6.775	583.	Vilazodone	-4.706
171.	Deferasirox	-6.747	584.	Sulfanilamide	-4.701
172.	Droperidol	-6.744	585.	Idebenone	-4.699
173.	Dexrazoxane	-6.733	586.	Fosfomycin tromethamine	-4.697
174.	Sparfloxacin	-6.724	587.	Simvastatin	-4.697
175.	Aniracetam	-6.700	588.	Batimastat	-4.694
176.	Anagrelide	-6.697	589.	Glyburide	-4.694
177.	Carbadox	-6.693	590.	Ranolazine	-4.693
178.	Gemcitabine	-6.691	591.	Alogliptin	-4.686
179.	Miglitol	-6.690	592.	Mirtazapine	-4.683
180.	Mecarbinate	-6.686	593.	Trimebutine	-4.678
181.	Floxuridine	-6.681	594.	Mianserin	-4.673
182.	Aminoglutethimide	-6.679	595.	Thiamine	-4.673
183.	Dexamethasone	-6.671	596.	Butoconazole	-4.658
184.	Diacerein	-6.664	597.	Benzbromarone	-4.657
185.	Mepiroxol	-6.663	598.	Nimodipine	-4.652
186.	Domperidone	-6.661	599.	Naftopidil	-4.643
187.	Besifloxacin	-6.648	600.	Brimonidine tartrate	-4.64
188.	Voglibose	-6.623	601.	Alibendol	-4.639
189.	Phenylbutazone	-6.614	602.	Rimantadine	-4.636
190.	Triamcinolone	-6.613	603.	Methyclothiazide	-4.622
191.	Lamivudine	-6.608	604.	Sulfamethazine	-4.619
192.	Nifenazone	-6.600	605.	Gabexate mesylate	-4.612
193.	Gatifloxacin	-6.584	606.	Dexmedetomidine	-4.610
194.	Adenine	-6.577	607.	Halobetasol propionate	-4.609
195.	Idoxuridine	-6.576	608.	Oxybutynin	-4.601
196.	Deferiprone	-6.574	609.	Tizanidine	-4.601
197.	Dextrose	-6.571	610.	Pranlukast	-4.600
198.	Tetracycline	-6.565	611.	Prazosin	-4.588
199.	Aminophylline	-6.547	612.	Fluconazole	-4.583
200.	Meprednisone	-6.547	613.	Varlitinib	-4.577
201.	Chlorzoxazone	-6.534	614.	Semagacestat	-4.575
202.	Lithocholic acid	-6.523	615.	Ethionamide	-4.567
203.	Dacarbazine	-6.510	616.	Halcinonide	-4.551
204.	Etravirine	-6.498	617.	Esmolol	-4.546
205.	Penciclovir	-6.495	618.	Dorzolamide	-4.544
206.	Betamipron	-6.494	619.	Melatonin	-4.527
207.	Tioxolone	-6.492	620.	Irbesartan	-4.524
208.	Bromfenac	-6.488	621.	Naratriptan	-4.522

209.	Furaltadone	-6.484	622.	Medetomidine	-4.520
210.	Cephalexin	-6.483	623.	Ritodrine	-4.497
211.	Sulfacetamide	-6.483	624.	Doxapram	-4.495
212.	Marbofloxacin	-6.469	625.	Aspartame	-4.480
213.	Veliparib	-6.465	626.	Olanzapine	-4.476
214.	Aztreonam	-6.460	627.	Trazodone	-4.469
215.	Bemegride	-6.458	628.	Vitamin A	-4.468
216.	Hyoscyamine	-6.455	629.	Estradiol valerate	-4.439
217.	Piperine	-6.446	630.	Choline	-4.438
218.	Teriflunomide	-6.444	631.	Eprazinone	-4.419
219.	D-Phenylalanine	-6.441	632.	Nepafenac	-4.415
220.	Aminothiazole	-6.435	633.	Clonidine	-4.413
221.	Mirabegron	-6.433	634.	Benzocaine	-4.409
222.	Xylose	-6.432	635.	Istradefylline	-4.407
223.	Artesunate	-6.430	636.	Eletriptan	-4.396
224.	Ganetespib	-6.430	637.	Ezetimibe	-4.394
225.	Albendazole	-6.428	638.	Prilocaine	-4.377
226.	Pirfenidone	-6.426	639.	Methimazole	-4.374
227.	Pyrazinamide	-6.426	640.	Oxybuprocaine	-4.347
228.	Adapalene	-6.422	641.	Valsartan	-4.328
229.	Carmofur	-6.422	642.	Nifuroxazide	-4.310
230.	Meloxicam	-6.421	643.	Rasagiline mesylate	-4.309
231.	Ronidazole	-6.417	644.	Verapamil	-4.306
232.	Tenoxicam	-6.416	645.	Phenacetin	-4.294
233.	Ipriflavone	-6.409	646.	Artemether	-4.293
234.	Tiopronin	-6.403	647.	Terazosin	-4.292
235.	Florfenicol	-6.401	648.	Trelagliptin	-4.289
236.	Mercaptopurine	-6.400	649.	Dofetilide	-4.285
237.	Bendamustine	-6.396	650.	Ebastine	-4.277
238.	Galanthamine	-6.392	651.	Clozapine	-4.271
239.	Acemetacin	-6.388	652.	Oxcarbazepine	-4.259
240.	Lomustine	-6.386	653.	Miconazole	-4.252
241.	Azathioprine	-6.384	654.	Sulconazle	-4.217
242.	Irsogladine	-6.384	655.	Ziprasidone	-4.192
243.	Dinitolmide	-6.383	656.	Quinine	-4.184
244.	Salbutamol	-6.381	657.	Camylofine	-4.178
245.	Divalproex	-6.379	658.	Oxaliplatin	-4.172
246.	Lafutidine	-6.371	659.	Betahistine	-4.112
247.	Serotonin	-6.371	660.	Cilnidipine	-4.109
248.	Danofloxacin mesylate	-6.370	661.	Mexiletine	-4.105
249.	Altretamine	-6.363	662.	Memantine	-4.102
250.	Gemfibrozil	-6.361	663.	Silodosin	-4.101
251.	Misoprostol	-6.360	664.	Bupivacaine	-4.100
252.	Canagliflozin	-6.353	665.	Ramelteon	-4.099
253.	Ozagrel	-6.342	666.	Diltiazem	-4.031
254.	Trifluridine	-6.331	667.	Fluorometholone	-4.026

255.	Pentoxifylline	-6.329	668.	Cysteamine	-4.011
256.	Vildagliptin	-6.329	669.	Moxonidine	-4.008
257.	Methacycline	-6.323	670.	Miconazole	-3.999
258.	Broxyquinoline	-6.321	671.	Apatinib	-3.998
259.	Amiloride	-6.317	672.	Roflumilast	-3.997
260.	Cephapirin	-6.317	673.	Lacidipine	-3.992
261.	Phenazopyridine	-6.313	674.	Tetrahydrozoline	-3.976
262.	Phenylephrine	-6.305	675.	Hydroxyzine	-3.970
263.	Baricitinib	-6.299	676.	Amorolfine	-3.968
264.	Azacitidine	-6.297	677.	Celecoxib	-3.967
265.	Triamterene	-6.296	678.	Ambroxol	-3.961
266.	Ftorafur	-6.296	679.	Nefopam	-3.958
267.	Chenodiol	-6.295	680.	Azacyclonol	-3.936
268.	5-Aminolevulinic acid	-6.281	681.	Protionamide	-3.906
269.	Antipyrine	-6.279	682.	Cinacalcet	-3.897
270.	Benzylpenicillin	-6.269	683.	Alfacalcidol	-3.891
271.	Lornoxicam	-6.267	684.	Isoconazole	-3.885
272.	Piroxicam	-6.266	685.	Eplerenone	-3.878
273.	Finasteride	-6.262	686.	Tripelennamine	-3.870
274.	Guaifenesin	-6.259	687.	Letrozole	-3.868
275.	Zanamivir	-6.255	688.	Ropivacaine	-3.859
276.	Thioguanine	-6.251	689.	Timolol maleate	-3.853
277.	Aceclidine	-6.242	690.	Mechlorethamine	-3.847
278.	Nefiracetam	-6.240	691.	Alizapride	-3.843
279.	Flumazenil	-6.237	692.	Tolbutamide	-3.841
280.	Amoxicillin	-6.232	693.	Clomipramine	-3.837
281.	Gliclazide	-6.226	694.	Lidocaine	-3.817
282.	Imatinib	-6.226	695.	Almotriptan	-3.812
283.	Methylthiouracil	-6.225	696.	Loxapine	-3.807
284.	Atovaquone	-6.212	697.	Tacrine	-3.805
285.	Tianeptine	-6.212	698.	Moclobemide	-3.791
286.	Difluoxacin	-6.209	699.	Asenapine	-3.790
287.	Oxytetracycline	-6.205	700.	Bufexamac	-3.778
288.	Praziquantel	-6.204	701.	Bisoprolol	-3.762
289.	Methoxsalen	-6.199	702.	Aminacrine	-3.743
290.	Telbivudine	-6.195	703.	Sulfamethizole	-3.723
291.	Pioglitazone	-6.187	704.	Sulfamethoxazole	-3.722
292.	Cetirizine	-6.186	705.	Fluoxetine	-3.702
293.	Ipratropium	-6.185	706.	Procaine	-3.696
294.	Clofarabine	-6.184	707.	Leflunomide	-3.691
295.	Bumetanide	-6.183	708.	Pramipexole.	-3.685
296.	Tropicamide	-6.181	709.	Sulfisoxazole	-3.678
297.	Alfuzosin	-6.180	710.	Metformin	-3.665
298.	Penicillamine	-6.176	711.	Primaquine	-3.665
299.	Ifosfamide	-6.175	712.	Tobramycin	-3.665
300.	Cyromazine	-6.174	713.	Ethacridine	-3.658

301.	Tolvaptan	-6.173	714.	Clorsulon	-3.632
302.	Nevirapine	-6.172	715.	Mepivacaine	-3.622
303.	Estriol	-6.162	716.	Cilostazol	-3.619
304.	Coumarin	-6.160	717.	Procyclidine	-3.599
305.	Mycophenolic acid	-6.159	718.	Amlodipine	-3.576
306.	Lincomycin	-6.155	719.	Thioridazine	-3.565
307.	Sulfameter	-6.154	720.	Levosimendan	-3.547
308.	Dinoprostone	-6.153	721.	Phenoxybenzamine	-3.545
309.	Lansoprazole	-6.145	722.	Cefdini	-3.511
310.	Sulphadimethoxine	-6.142	723.	Mubritinib	-3.510
311.	Homatropine	-6.139	724.	Phenformin	-3.505
312.	Homatropine	-6.132	725.	Reboxetine	-3.457
313.	Bisacodyl	-6.117	726.	Flunarizine	-3.453
314.	Pregnenolone	-6.112	727.	Dapoxetine	-3.452
315.	Ganciclovir	-6.110	728.	Ambrisentan	-3.435
316.	Sulfapyridine	-6.095	729.	Naphazoline	-3.426
317.	Empagliflozin	-6.092	730.	Cimetidine	-3.410
318.	Thiamphenicol	-6.090	731.	Repaglinide	-3.406
319.	Pyridoxine	-6.089	732.	Orlistat	-3.396
320.	Chlormezanone	-6.088	733.	Levobetaxolol	-3.390
321.	Pravastatin	-6.085	734.	Vortioxetine	-3.359
322.	Mequinol	-6.081	735.	Trimipramine	-3.327
323.	Bicalutamide	-6.075	736.	Pizotifen	-3.313
324.	Dichlorphenamide	-6.074	737.	Rilpivirine	-3.300
325.	Pazopanib	-6.066	738.	Mifepristone	-3.292
326.	Etofibrate	-6.057	739.	Sulfathiazole	-3.292
327.	Resveratrol	-6.048	740.	Atomoxetine	-3.286
328.	Anisindione	-6.042	741.	Cyproheptadine	-3.273
329.	Meptazinol	-6.041	742.	Chlorpheniramine	-3.226
330.	Pramiracetam	-6.037	743.	Dibucaine	-3.224
331.	Silibinin	-6.037	744.	Imipramine	-3.211
332.	Carbidopa	-6.024	745.	Clopidogrel	-3.208
333.	Methocarbamol	-6.021	746.	Disopyramide	-3.194
334.	Carbimazole	-6.009	747.	Phenothiazine	-3.194
335.	Bentiromide	-5.997	748.	Guanethidine	-3.175
336.	Enalapril	-5.997	749.	Vitamin D2	-3.163
337.	Racecadotril	-5.996	750.	Tolazoline	-3.152
338.	Cloxacillin	-5.991	751.	Dyclonine	-3.137
339.	Torsemide	-5.991	752.	Isradipine	-3.128
340.	Cytisine	-5.986	753.	Desloratadine	-3.126
341.	Chlorocresol	-5.983	754.	Brompheniramine	-3.098
342.	Hydralazine	-5.983	755.	Flutamide	-3.097
343.	Ampicillin	-5.977	756.	Trospium	-3.069
344.	Nafcillin	-5.966	757.	Tolperisone	-3.063
345.	Benserazide	-5.965	758.	Adiphenine	-3.049
346.	Azilsartan	-5.960	759.	Risperidone	-3.030

347.	Formestane	-5.958	760.	Cyclizine	-3.018
348.	Glimepiride	-5.950	761.	Oxacillin	-3.013
349.	Edaravone	-5.947	762.	Chlorpromazine	-3.005
350.	Vidarabine	-5.946	763.	Articaine	-2.999
351.	Ampicillin	-5.924	764.	Fluvoxamine	-2.996
352.	Tideglusib	-5.921	765.	Orphenadrine	-2.990
353.	Rolipram	-5.917	766.	Xylometazoline	-2.960
354.	Laquinimod	-5.915	767.	Butenafine	-2.952
355.	Zolmitriptan	-5.915	768.	Sertraline	-2.922
356.	Avobenzone	-5.910	769.	Methenamine	-2.911
357.	Norethindrone	-5.909	770.	Benzethonium	-2.898
358.	Doxofylline	-5.905	771.	Guanabenz	-2.883
359.	Brinzolamide	-5.902	772.	Maprotiline	-2.879
360.	Cyclophosphamide	-5.902	773.	Ticlopidine	-2.878
361.	Exemestane	-5.901	774.	Doxylamine	-2.876
362.	Dropropizine	-5.900	775.	Thiotepa	-2.851
363.	Dexlansoprazole	-5.897	776.	Mitotane	-2.846
364.	Zonisamide	-5.897	777.	Clotrimazole	-2.828
365.	Estrone	-5.887	778.	Prednisolone	-2.826
366.	D-Mannitol	-5.886	779.	Diphenhydramine	-2.818
367.	Granisetron	-5.880	780.	Diphemanil methylsulfate	-2.813
368.	Primidone	-5.879	781.	Pheniramine	-2.796
369.	Cycloserine	-5.871	782.	Benztropine	-2.754
370.	Sotalol	-5.852	783.	Proparacaine	-2.736
371.	Estradiol	-5.829	784.	Xylazine	-2.698
372.	Methscopolamine	-5.828	785.	DicycloMine	-2.668
373.	Neostigmine	-5.803	786.	Diphenylpyraline	-2.655
374.	Pyrimethamine	-5.797	787.	Trifluoperazine	-2.655
375.	Rosiglitazone	-5.789	788.	Chlorprothixene	-2.638
376.	Ketanserin	-5.784	789.	Duloxetine	-2.631
377.	Nitazoxanide	-5.782	790.	Betamethasone	-2.570
378.	Tretinoin	-5.779	791.	Meclizine	-2.556
379.	Dapagliflozin	-5.778	792.	Terbinafine	-2.548
380.	Flumethasone	-5.774	793.	Amitriptyline	-2.531
381.	Meglumine	-5.769	794.	Amantadine	-2.523
382.	Fasudil	-5.767	795.	Proadifen	-2.523
383.	Alprostadil	-5.761	796.	Pergolide	-2.505
384.	Nicorandil	-5.761	797.	Pramoxine	-2.496
385.	Amprolium	-5.750	798.	Vorinostat	-2.484
386.	Budesonide	-5.748	799.	Propafenone	-2.453
387.	(-)-Huperzine A	-5.747	800.	Tetracaine	-2.403
388.	S-(+)-Rolipram	-5.742	801.	Mepenzolate romide	-2.396
389.	Sorbitol	-5.735	802.	Allylthiourea	-2.392
390.	Griseofulvin	-5.722	803.	Vitamin D3	-2.374
391.	Resminostat	-5.721	804.	Megestrol	-2.365
392.	Didanosine	-5.713	805.	Oxethazaine	-2.361

393.	Scopolamine	-5.713	806.	Benzydamine	-2.336
394.	Ethoxzolamide	-5.710	807.	Hexamethonium	-2.240
395.	Pimobendan	-5.710	808.	Tolnaftate	-2.236
396.	Methylprednisolone	-5.705	809.	Tolterodine	-2.223
397.	Metronidazole	-5.701	810.	Succinylcholine	-2.220
398.	Malotilate	-5.701	811.	Loteprednol etabonate	-2.196
399.	Sulfamerazine	-5.700	812.	Bromhexine	-2.042
400.	Fluocinolone	-5.695	813.	Terbinafine	-1.989
401.	Fenspiride	-5.692	814.	Clofazimine	-1.985
402.	Ascorbic acid	-5.686	815.	Ethynodiol	-1.937
403.	Carbachol	-5.681	816.	Arecoline	-1.852
404.	Rofecoxib	-5.678	817.	Nizatidine	-1.732
405.	Bimatoprost	-5.676	818.	Bephenium hydroxynaphthoate	-1.696
406.	Cortisone acetate	-5.672	819.	Clemastine	-1.679
407.	Toltrazuril	-5.665	820.	Domiphen	-1.607
408.	Ramipril	-5.660	821.	Ranitidine	-1.601
409.	Clorprenaline	-5.660	822.	Calcifediol	-1.521
410.	Chloramphenicol	-5.655	823.	Cetylpyridinium	-1.443
411.	Triclabendazole	-5.645	824.	Ethambutol	-0.810
412.	Artemisinin	-5.642	825.	Alverine	-0.158
413.	Lamotrigine	-5.630			

Table S2. Standard precision (SP) docking parameters of drugs shortlisted on the basis of HTVS score (≤ -7.0 kcal mol^{-1}).

S. No.	Name of drug	Docking score (kcal mol^{-1})	Glide g-score (kcal mol^{-1})	Glide e-model (kcal mol^{-1})
1.	Foscarnet	-9.123	-9.129	-108.829
2.	Raltitrexed	-9.043	-9.107	-140.610
3.	Etidronate	-8.999	-9.245	-138.567
4.	Risedronate	-8.917	-9.105	-135.810
5.	Fludarabine	-8.751	-8.843	-129.947
6.	Epalrestat	-8.622	-8.622	-93.373
7.	Pamidronate	-8.614	-8.724	-117.857
8.	Ethamsylate	-8.555	-8.556	-78.285
9.	Gimeracil	-8.367	-8.367	-69.806
10.	Ticarcillin	-8.352	-8.352	-105.343
11.	Carbenicillin	-8.273	-8.273	-104.280
12.	Alendronate	-8.271	-8.470	-123.768
13.	Pasiniazid	-8.240	-8.241	- 77.361
14.	Procodazole	-8.225	-8.266	- 87.745
15.	Zalcitabine	-8.215	-8.215	- 73.739
16.	Mizoribine	-8.207	-8.220	- 90.982
17.	Nalidixic acid	-8.205	-8.250	- 84.175
18.	Carboplatin	-8.192	-9.216	-110.918
19.	Methotrexate	-8.132	-8.197	-116.799

20.	Sodium butyrate	-8.125	-8.128	-70.709
21.	Ibandronate	-8.083	-8.174	-122.715
22.	Tenofovir	-8.040	-8.622	-121.062
23.	Acipimox	-8.023	-8.027	-72.998
24.	Fosbretabulin	-7.996	-8.060	-112.705
25.	Biotin	-7.989	-7.993	-89.587
26.	Aspirin	-7.972	-7.972	-81.327
27.	Niacin	-7.940	-7.949	-72.803
28.	Phthalylsulfacetamide	-7.925	-7.937	-106.29
29.	Pircetam	-7.895	-7.895	-57.812
30.	Tasisulam	-7.864	-7.865	-91.296
31.	Zileuton	-7.855	-7.873	-62.328
32.	Cytarabine	-7.831	-7.831	-73.499
33.	Sulbactam	-7.829	-7.829	-75.507
34.	Furosemide	-7.827	-7.827	-90.415
35.	Pidotimod	-7.806	-7.806	-90.853
36.	Rebamipide	-7.782	-7.782	-94.894
37.	Isosorbide	-7.768	-7.768	-54.515
38.	Benzoic acid	-7.767	-7.768	-71.404
39.	Flumequine	-7.762	-7.835	-87.148
40.	Mesna	-7.749	-7.751	-71.850
41.	Dimesna	-7.691	-7.691	-89.041
42.	Triflusal	-7.685	-7.685	-79.680
43.	Nateglinide	-7.683	-7.683	-94.275
44.	Tranilast	-7.680	-7.680	-90.622
45.	Cinoxacin	-7.670	-7.697	-81.509
46.	Cidofovir	-7.664	-8.217	-116.596
47.	Zaltoprofen	-7.655	-7.656	-83.932
48.	Flucytosine	-7.642	-7.642	-50.160
49.	Sodium ascorbate	-7.613	-7.613	-59.354
50.	Tranexamic acid	-7.602	-7.602	-67.713
51.	Oxiracetam	-7.595	-7.595	-57.234
52.	Entecavir	-7.585	-7.585	-65.147
53.	Valganciclovir	-7.565	-7.779	-91.223
54.	Cinchophen	-7.559	-7.560	-85.270
55.	Orotic acid	-7.556	-7.556	-67.307
56.	Captopril	-7.543	-7.543	-76.978
57.	Sodium picosulfate	-7.540	-7.552	-86.858
58.	Olsalazine	-7.520	-7.520	-83.943
59.	Olopatadine	-7.508	-7.508	-88.016
60.	Chromocarb	-7.503	-7.503	-68.118
61.	Sodium gluconate	-7.469	-7.469	-82.280
62.	Taurine	-7.467	-7.468	-52.865
63.	Dyphylline	-7.460	-7.460	-65.603
64.	Indomethacin	-7.456	-7.457	-84.428
65.	Felbamate	-7.452	-7.452	-68.384

66.	Sulindac	-7.444	-7.444	-77.570
67.	Ketoprofen	-7.437	-7.438	-80.552
68.	Piromidic acid	-7.428	-7.458	-83.963
69.	Ciclopirox	-7.383	-7.383	-70.967
70.	Meropenem	-7.378	-7.407	-86.425
71.	Aicar	-7.362	-7.362	-60.074
72.	Etodolac	-7.352	-7.353	-77.318
73.	Tolmetin	-7.337	-7.338	-74.503
74.	Inosine	-7.279	-7.279	-61.930
75.	Isoniazid	-7.265	-7.266	-48.708
76.	Adenosine	-7.251	-7.251	-62.475
77.	Suprofen	-7.244	-7.244	-78.898
78.	Ribavirin	-7.238	-7.238	-59.295
79.	Oxfendazole	-7.234	-7.434	-71.321
80.	Uridine	-7.177	-7.179	-59.163
81.	Enalaprilat	-7.156	-7.282	-75.969
82.	Glafenine	-7.151	-7.732	-75.676
83.	Clinofbrate	-7.134	-7.134	-96.866
84.	Erdosteine	-7.130	-7.130	-76.878
85.	Oxaprozin	-7.128	-7.129	-83.109
86.	Lonidamine	-7.122	-7.122	-83.615
87.	Streptozocin	-7.109	-7.109	-59.470
88.	Doxifluridine	-7.103	-7.140	-62.158
89.	Pefloxacin mesylate	-7.087	-7.248	-81.811
90.	Clofibric acid	-7.068	-7.068	-73.156
91.	Enoxacin	-7.065	-7.112	-79.847
92.	Flufenamic acid	-7.058	-7.059	-77.335
93.	Mefenamic acid	-7.047	-7.048	-78.325
94.	Naproxen	-7.029	-7.030	-72.750
95.	Ibuprofen	-7.004	-7.005	-72.774
96.	Diclofenac	-6.996	-6.997	-80.302
97.	Niflumic acid	-6.966	-6.996	-77.842
98.	Bindarit	-6.937	-6.937	-85.039
99.	Sodium phenylbutyrate	-6.901	-6.903	-69.181
100.	Sarafloxacin	-6.876	-6.947	-86.362
101.	Flunixin meglumine	-6.875	-6.905	-75.318
102.	Dopamine	-6.838	-6.838	-45.573
103.	Febuxostat	-6.753	-6.754	-80.477
104.	Ciprofibrate	-6.695	-6.695	-74.465
105.	(R)-Baclofen	-6.685	-6.685	-61.289
106.	L-Carnitine	-6.684	-6.684	-60.776
107.	Nitrofurazone	-6.672	-6.672	-48.876
108.	Noradrenaline bitartrate	-6.426	-6.444	-48.671
109.	Methyldopa	-6.421	-6.421	-56.030
110.	Enrofloxacin	-6.337	-6.538	-70.118
111.	Lisinopril	-6.336	-6.458	-88.749

112.	Omeprazole	-6.278	-6.372	-62.997
113.	DL-Carnitine	-6.247	-6.247	-57.517
114.	Dihydroartemisinin	-6.244	-6.244	-43.018
115.	Flopropione	-5.918	-6.354	-57.484
116.	Diclazuril	-5.888	-6.285	-72.389
117.	Tolcapone	-5.831	-5.886	-61.033
118.	Chloroxine	-5.536	-5.670	-48.103
119.	Chlorothiazide	-4.789	-5.103	-56.436
